# Impact of Providing a Personalized Data Dashboard on Ecological Momentary Assessment Compliance Among College Students Who Use Substances: Pilot Microrandomized Trial

**DOI:** 10.2196/60193

**Published:** 2024-12-05

**Authors:** Ashley Linden-Carmichael, Samuel W Stull, Danny Wang, Sandesh Bhandari, Stephanie T Lanza

**Affiliations:** 1 The Edna Bennett Pierce Prevention Research Center The Pennsylvania State University University Park, PA United States; 2 Department of Biobehavioral Health The Pennsylvania State University University Park, PA United States

**Keywords:** ecological momentary assessment, data dashboard, study compliance, substance use, substance use behavior, college student, alcohol, cannabis, cannabis use, personalized data dashboard, EMA protocol, EMA, health behaviors, survey, compliance, self-reported

## Abstract

**Background:**

The landscape of substance use behavior among young adults has observed rapid changes over time. Intensive longitudinal designs are ideal for examining and intervening in substance use behavior in real time but rely on high participant compliance in the study protocol, representing a significant challenge for researchers.

**Objective:**

This study aimed to evaluate the effect of including a personalized data dashboard (DD) in a text-based survey prompt on study compliance outcomes among college students participating in a 21-day ecological momentary assessment (EMA) study.

**Methods:**

Participants (N=91; 61/91, 67% female and 84/91, 92% White) were college students who engaged in recent alcohol and cannabis use. Participants were randomized to either complete a 21-day EMA protocol with 4 prompts/d (EMA Group) or complete the same EMA protocol with 1 personalized message and a DD indicating multiple metrics of progress in the study, delivered at 1 randomly selected prompt/d (EMA+DD Group) via a microrandomized design. Study compliance, completion time, self-reported protocol experiences, and qualitative responses were assessed for both groups.

**Results:**

Levels of compliance were similar across groups. Participants in the EMA+DD Group had overall faster completion times, with significant week-level differences in weeks 2 and 3 of the study (*P*=.047 and *P*=.03, respectively). Although nonsignificant, small-to-medium effect sizes were observed when comparing the groups in terms of compensation level (*P*=.08; Cohen *w*=0.19) and perceived burden (*P*=.09; Cohen *d*=-0.36). Qualitative findings revealed that EMA+DD participants perceived that seeing their progress facilitated engagement. Within the EMA+DD Group, providing a DD at the moment level did not significantly impact participants’ likelihood of completing the EMA or completion time at that particular prompt (all *P*>.05), with the exception of the first prompt of the day (*P*=.01 and *P*<.001).

**Conclusions:**

Providing a DD may be useful to increase engagement, particularly for researchers aiming to assess health behaviors shortly after a survey prompt is deployed to participants’ mobile devices.

**International Registered Report Identifier (IRRID):**

RR2-10.2196/57664

## Introduction

The landscape of substance use behavior among college-attending young adults (aged approximately 18 to 25 years) has changed in recent years. During the last 10 years, the prevalence of past-month drinking, daily drinking, and heavy episodic drinking (4+ or 5+ drinks in an occasion for female or male individuals, respectively) have declined in this age group, although rates remain high, with 31% of young adults aged 19-30 years reporting past-2-week heavy episodic drinking [1]. Conversely, rates of daily cannabis use (20+ use occasions in the past 30 days) are at all-time highs, with 11% of young adults reporting daily use [1]. Pacing with increases in cannabis use in recent years, young adults are also increasingly reporting co-use of alcohol and cannabis use, with approximately one-quarter reporting past-year simultaneous use or use of both substances so that the effects overlap [2]. Many young adults experience acute negative consequences related to heavy and frequent alcohol or cannabis use, including blacking out, social or interpersonal concerns, or adverse physical effects [3]. Moreover, many young adults meet the criteria for past-year alcohol use disorder (16.4%) and cannabis use disorder (16.5%) [4].

Daily diary and ecological momentary assessment (EMA) designs have become ubiquitous to health researchers assessing fine-grained daily or momentary predictors and outcomes, such as alcohol and cannabis use behavior, as well as for delivering timely intervention content based on participant responses (eg, mood and social setting) [5,6]. However, they often require participants to initiate action or respond to a prompt. For example, studies deploying intervention content or developing “decision rules” for delivery typically rely heavily on high participant compliance in completing mobile surveys soon after each prompt is sent. In addition, just-in-time interventions may involve detecting and disseminating messages during sensitive time windows, for example, delivering time-sensitive momentary feedback on a participant’s speed of alcohol consumption during the beginning of a drinking episode may rely on the participant self-reporting the amount of alcohol they have consumed thus far and their level of perceived impairment. EMA protocols assessing substance use behavior may also rely on participants completing surveys on days when certain behaviors are more common, such as on weekend days [7], and EMA protocols detecting higher-risk but infrequent behavior such as simultaneous alcohol and cannabis use also rely on high participant compliance [8]. Unfortunately, compliance rates for EMA protocols average around 75% [9], and young adults are less likely to complete survey prompts on weekends or heavy substance use days [7]. Due to the need to balance high compliance with minimizing participant burden and disruption to their daily lives, EMA protocols may not be adequately capturing behavior when health risks of interest are more likely. Low compliance can reduce our understanding of the etiology of substance use behaviors in daily life and, by missing responses during high-risk time windows, moments of greatest risk when intervention content is most needed may be less likely to be detected.

Best practices for increasing participant compliance in diary and EMA designs have received much attention, and findings have been inconsistent with regard to features such as length of survey, assessment frequency, incentive structure, and sampling schedule [9-12]. Recent efforts have been made to increase participant engagement and thereby compliance with EMA study protocols [13,14]. One promising approach is to personalize the interface, such as using their name at each prompt. Recent EMA trials have shown that personalization can have an overall and time-varying impact on participant compliance throughout the protocol [15]; however, similar to other metrics of compliance, providing personalized messages has yielded differential findings. Some EMA work has found that providing summaries of participant compliance improved compliance [16], while others found no such difference [11,17]. Supported by behavioral economics and motivational theories including agency and intrinsic motivational theory (for a review, see [18]) incorporating game design elements or “gamification” may further increase participant engagement although research in this area has been limited in EMA studies [19,20]. One useful personalized approach that targets engagement may be to provide participants with a data dashboard (DD) to view their progress in an EMA protocol, the amount of money they have received thus far, and their progress toward a monetary bonus for high compliance. Whether such a DD can increase compliance in an EMA protocol overall and across time, as well as whether a DD has immediate impacts on participant compliance in a survey prompt, await empirical investigation.

As described in our team’s study protocol paper [21], Project ENGAGE involved the creation of a DD that delivered real-time feedback on the participant’s study progress in terms of number of surveys completed, amount of compensation earned, and progress toward a high-compliance bonus. Participants were randomly assigned to either (1) an “EMA as usual” (EMA Group) protocol, in which they participated in a 21-day EMA study with 4 survey prompts per day, or (2) an “EMA + DD” (EMA+DD Group) protocol, in which they participated in the same study design but also received a personalized message with a DD once per day. Using a microrandomized trial [22] design (ie, an experimental design in which participants are randomized to receive different types of interventions), participants in this group were randomized each day to which survey prompt of the day would include a personalized message and embedded DD showing up-to-date information about their progress in the study protocol.

The goal of this study was to report on the findings from the protocol reported by Lanza et al [21] to evaluate the effect of including a personalized DD in a text-based survey prompt on study compliance outcomes among college students participating in the EMA protocol. Specifically, this study had 3 aims. First, we aimed to evaluate the overall effect of a daily DD by estimating group differences in overall compliance, average completion time (if completed), and subjective experiences of the EMA protocol between the 2 groups. Second, we aimed to estimate group differences in compliance across weeks in the study (overall compliance; average completion time across weeks 1, 2, and 3) to determine whether the effects varied with time in the study. Third, within the EMA+DD Group, using a microrandomized trial, we aimed to examine the momentary impact of receiving a DD on survey completion (yes/no) and survey completion time. Further, we aimed to examine whether the association between receiving a DD (yes/no) on study outcomes (survey completion and completion time) was moderated by the time of day the DD was sent (ie, 11 AM, 2 PM, 5 PM, and 8 PM).

## Methods

### Participants and Procedures

College students from a public university in the Northeastern region of the United States who previously participated in a campus-wide survey in February and March 2023, agreed to be contacted about future research opportunities, and reported past month alcohol use or lifetime cannabis use on the initial survey were provided information about this study. Interested participants completed an eligibility screener and, if eligible, were provided the study consent form. To be eligible, participants must have been between 18 and 25 years old and have reported alcohol and cannabis use in the past 30 days. To facilitate the automation of this complex design, participants were also required to use an iPhone with iOS 12 (operating system) or above to complete surveys. A total of 411 students were invited to the study. Of those invited, 200 students replied and were emailed survey screeners, of which 101 students were deemed eligible. It should be noted that although the response rate was fairly low (200/411, 49%); for context, the recruitment window was only open until the desired enrollment was filled. Thus, enrollment was open for only 11 days. A total of 92 students participated in the study and 1 student withdrew, yielding a final analytic sample of 91 participants. Participants primarily identified as female (n=61, 67%), White (n=84, 92%), and non-Hispanic (n=81, 89%); not affiliated with a fraternity or sorority (n=68, 75%); and residing off-campus (n=64, 70%). Table 1 contains a full summary of demographic characteristics.

**Table 1:** Participant demographics (n=91).

Demographics and response options		Values, n (%)
**Sex**		
	Male	30 (0.33)
	Female	61 (0.67)
**Gender**		
	Man	29 (0.32)
	Woman	59 (0.65)
	Nonbinary	2 (0.02)
	Prefer not to say	1 (0.01)
**Hispanic**		
	Yes	10 (0.11)
	No	81 (0.89)
**Race**		
	Asian	2 (0.02)
	Black	1 (0.01)
	White	84 (0.92)
	Multiracial	4 (0.04)
**Greek**		
	Yes	23 (0.25)
	No	68 (0.75)
**College standing**		
	First year	14 (0.15)
	Second year	23 (0.25)
	Third year	25 (0.27)
	Fourth year	27 (0.30)
	Fifth year	2 (0.02)
**Residence**		
	Parent’s or relative’s home	1 (0.01)
	College dorm or residence hall	23 (0.25)
	House, apartment, or room	64 (0.70)
	Fraternity or sorority house	2 (0.02)
	Prefer not to say	1 (0.01)

**Figure 1:**
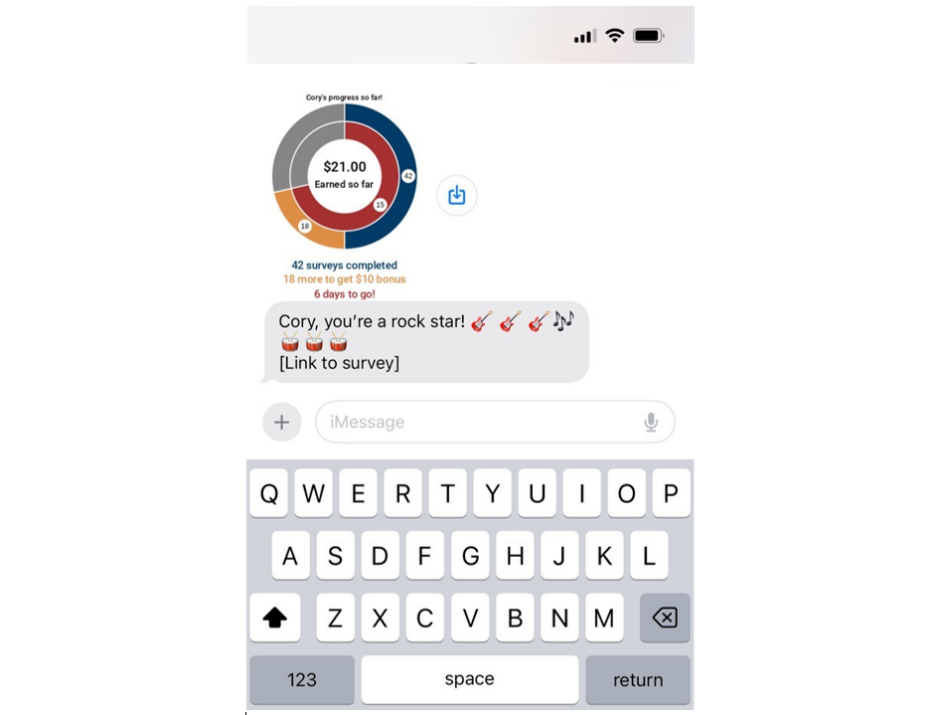
Screenshot of sample data dashboard from Project ENGAGE study team.

Once participants provided informed consent and were deemed eligible for the study, they were asked to complete a web-based baseline survey via REDCap (Research Electronic Data Capture; Vanderbilt University) that took approximately 15-20 minutes. Participants were then randomized to one of two conditions: (1) a standard EMA protocol (4 surveys/d for 21 consecutive days) with standard text messages pushed at each prompt to complete the survey (EMA Group), or (2) the same protocol as in the EMA Group, plus a personalized message and DD reflecting their current progress in the study (EMA+DD Group) at 1 randomly selected prompt/d. Figure 1 shows a sample DD delivered via text message.

EMA survey prompts were provided via automated text messages (via REDCap) for all participants at the same times each day: 11 AM, 2 PM, 5 PM, and 8 PM for 21 consecutive days (15 weekdays and 6 weekend days). EMA surveys took approximately 1 minute to complete, and participants were allotted 60 minutes to complete the survey, after which the personalized link would expire. Reminder texts were not provided, given our focus on short-term compliance outcomes. The DD was provided at 1 randomly selected text message occasion per day. To do this, REDCap worked in conjunction with a private server implemented by the research team. The server was used to monitor the generation of EMA surveys, with a script then calculating the information needed to populate the current DD. The server provided the DD at 1 randomly selected prompt per day to the EMA+DD Group. Participants could earn up to US $67 via an e-gift card for full participation in the study. Specifically, participants were compensated US $10 for the baseline survey, US $0.50/daily survey, a US $10 bonus if they completed 70+ out of the 84 surveys, and US $5 for completing a brief exit survey. Additional details regarding the study can be found in the protocol paper (see Lanza et al [21]).

### Measures

In preparation for modeling the study outcomes, we first coded 2 variables at each prompt. At each text message occasion, a binary indicator was coded 1 if participants completed the survey within 60 minutes of receiving a prompt, and 0 otherwise. Additionally, a continuous indicator of completion time was coded as the number of minutes between receiving a prompt and completing the survey, conditional on completing the survey at that prompt.

Four primary outcome variables were examined as a function of the intervention group (EMA Only vs EMA+DD). First, overall study compliance was calculated as a person-level variable by summing the binary indicator of study completion across the 84 prompts. Second, average completion time was calculated as an individual’s average completion time (ie, time between survey prompt and submitting that survey), conditional on completing that survey (ie, if the binary compliance indicator =1 at that prompt). Third, self-reported protocol experiences were assessed once for each participant at the exit survey with 7 quantitative variables (eg, “How personalized did you feel the text messages were for you?”; Table 2 contains the complete list of questions). Fourth, subjective experiences were assessed as part of the exit survey, with open-ended questions slightly modified between the 2 groups to assess experiences with each condition; Table 3 contains the complete list of questions.

**Table 2:** Intervention group differences in self-reported experiences with the study protocol, assessed at exit survey (day 21).

Subjective experience		EMA^a^-Only group (n=43)	EMA+DD^b^ group (n=48)	*t* test (*df*)	Chi-square (*df*)	*P* value	Cohen *d* or Cohen *w*
**(1) How personalized did you feel the text messages were for you? (** **1=not at all personalized to me to 7=very personalized to me** **), mean (SD)**		3.5 (1.6)	4.3 (1.4)	2.46 (85)	—^c^	.02	0.52
**(2) Overall, how easy was it to complete the surveys on your iPhone? (1=very difficult to 7=very easy), mean (SD)**		6.4 (0.7)	6.5 (1.0)	0.15 (86)	—	.88	0.03
**(3) Overall, how engaged or motivated did you feel to complete the surveys? (1=not at all engaged to 7=very engaged), mean (SD)**		5.0 (1.4)	5.0 (1.3)	–0.32 (86)	—	.75	–0.06
**(4) Overall, how burdensome was it to complete the surveys on your iPhone? (1=not burdensome at all to 7=very burdensome), mean (SD)**		3.3 (1.5)	2.7 (1.5)	–1.70 (86)	—	.09	–0.36
**(5) How many days into the study did you begin to get tired of completing the surveys? (out of those who said they did get tired)** **, mean (SD)**		11.1 (4.8)	11.3 (3.7)	0.15 (65)	—	.88	0.04
	Number of participants who said they did not get tired of completing the surveys during the study, n (%)	8 (18.6)	13 (27.1)	—	0.50 (1)	.48	0.07
**(6) Were there certain surveys that were typically more difficult to complete on-time than others?** **n (%)**							
	Check all that apply: Weekend	18 (41.9)	23 (47.9)	—	0.14 (1)	.72	0.04
	Check all that apply: Weekdays	3 (7.0)	5 (10.4)	—	0.04 (1)	.84	0.02
	Check all that apply: Any particular survey time	29 (67.4)	35 (72.9)	—	0.12 (1)	.73	0.04
	Check all that apply: 11 AM	6 (14.0)	11 (22.9)	—	0.68 (1)	.41	0.09
	Check all that apply: 2 PM	7 (16.3)	6 (12.5)	—	0.05 (1)	.83	0.02
	Check all that apply: 5 PM	10 (23.3)	12 (25.0)	—	0.00 (1)	.99	0.00
	Check all that apply: 8 PM	12 (27.9)	17 (35.4)	—	0.29 (1)	.59	0.06
**(7) Was the compensation level adequate based on the amount of time you spent completing these surveys? (yes), n (%)**		33 (84.6)	43 (97.7)	—	3.06 (1)	.08	0.19

^a^EMA: ecological momentary assessment.

^b^DD: data dashboard.

^c^Not applicable.

**Table 3:** Open-ended responses regarding the general design of the data dashboard (ecological momentary assessment + data dashboard group only).

General theme			Example quote
**(1) What did you like about the data dashboard?**			
	Viewing progress (n=14)	“I loved seeing my progress and LOVED that it gave me a countdown”	
	Easy to understand/liked the design (n*=*13)	“Showed completion in a digital metric”	
	Helped them stay engaged (n=3)	“It showed me how much I had left to do and motivated me to keep completing the surveys”	
	Seeing money they had earned so far (n=3)	“It showed me how much I was making”	
**(2) What did you dislike about the data dashboard?**			
	Confusing/information could be clearer (n=6)	“Took me a second to understand it”	
	Wanting more access to it (n=3)	“I want constant access to it”	
	Disliked information provided (n=2)	“I didn’t like seeing how many days I still had left”	
	Functionality (n=1)	“It didn’t always load properly for me”	
	Visual appeal (n=1)	“Boring colors”	
**(3) Which features were most incentivizing?**			
	Amount earned so far (n=21)	“I like seeing how much I had earned so far!”	
	Progress in the study (n=14)	“Knowing how many days were left”	
	Bonus (n=12)	“Telling me how many more surveys I needed to complete before I earned an extra $10”	
	General design	“Coloured visual wheel not words”	

### Analytic Plan

Descriptive statistics were calculated for all demographic variables. Outcome measures were then calculated as described above. For exploratory analyses, we also calculated overall study compliance for each person within a week and within a day. To address aim 1, Poisson regression was used to compare group differences in person-level compliance (person-level count of completed prompts as outcome variable). The group differences in other person-level quantitative variables were tested using *t* tests (for continuous outcomes) and chi-square tests (for binary outcomes). Responses to open-ended questions about participants’ subjective experiences were summarized by identifying common descriptive themes that emerged across responses. Responses were analyzed descriptively given the small sample size with short, descriptive responses. The first author identified common themes and reached a consensus with coauthors regarding categorization.

Aim 2 analyses focused on study compliance and completion time, calculated within a week and within a day to examine possible time trends in differences across groups. To examine differences across weeks 1 through 3 of the study, repeated measures ANOVAs were specified to model an outcome as a function of the group, week in study (represented by 2 dummy-coded variables with week 1 serving as the reference group), and the group-by-study week interactions. Significant interaction terms were probed to interpret specific differences across weeks. Differences in daily compliance and daily completion time were examined graphically.

To address aim 3, we used generalized estimating equations to specify 2 models using all 84 time points. For Model 1, the outcome was moment-level survey completion; for Model 2, the outcome was completion time, given that the EMA was completed within 60 minutes. Analyses included only individuals in the EMA+DD Group, and both models included the following predictors: receiving a DD at that moment (reference=none), prompt at which DD was provided (reference=11 AM), and the interaction between moment-level DD and prompt of the day. This analysis follows recommended procedures to analyze data from a microrandomized trial [22]. All quantitative analyses were conducted in RStudio (version 4.3.0; RStudio Team).

### Ethical Considerations

The institutional review board at The Pennsylvania State University approved this study (STUDY00021945). Participants provided written informed consent before participation.

## Results

### Aim 1: EMA Versus EMA+DD Group Comparisons

Overall, out of a possible 1911 days of data and 7644 survey prompts delivered (4/d for 21 days), participants completed 5931 (77%) EMAs. Participants in the EMA+DD group completed a mean of 64.5 (SD 15.7) EMAs, and individuals in the EMA group completed a mean of 65.1 (SD 13.5); this difference was nonsignificant with a small effect (t_88.9_=0.172, *P*=.86; *d*=0.04). Of the EMAs completed, however, participants in the EMA+DD Group completed the survey significantly faster with a medium effect size (EMA Group: mean 881.2, SD 323.4 s vs EMA+DD Group: mean 738.8, SD 272 s; t_82.5_=–2.26, *P*=.03; *d*=–0.48).

Group comparisons of subjective experiences are shown in Table 2. Overall, participants in the EMA+DD Group were significantly (with a medium-sized effect) more likely to perceive the text messages as personalized than participants in the EMA Group. Although nonsignificant, 13% more participants in the EMA+DD Group perceived the compensation level to be adequate (33/39=84.6% in the EMA group, vs. 43/44=97.7% in the EMA+DD group), and there was a small-to-medium effect size difference (*P*=.09; Cohen *d*=-0.36) in perceiving the protocol as less burdensome.

With regard to qualitative participant feedback about the use of the DD within the EMA+DD Group, participants noted that they liked seeing their progress, that it was easy to understand or they liked the design, that it helped them stay engaged, and that they liked seeing the money they had earned so far. Regarding dislikes, some participants noted that it was confusing or that the information could be clearer, wanted more access to it, and disliked the information provided and the features of the visual appeal. Finally, participants were asked which features of the DD graph were most incentivizing. Participants positively responded to the amount earned so far, their progress in the study, the bonus specifically, and the general design. Example quotes for each theme are provided in Table 3.

### Aim 2: Group Differences Across Weeks in Study

**Figure 2:**
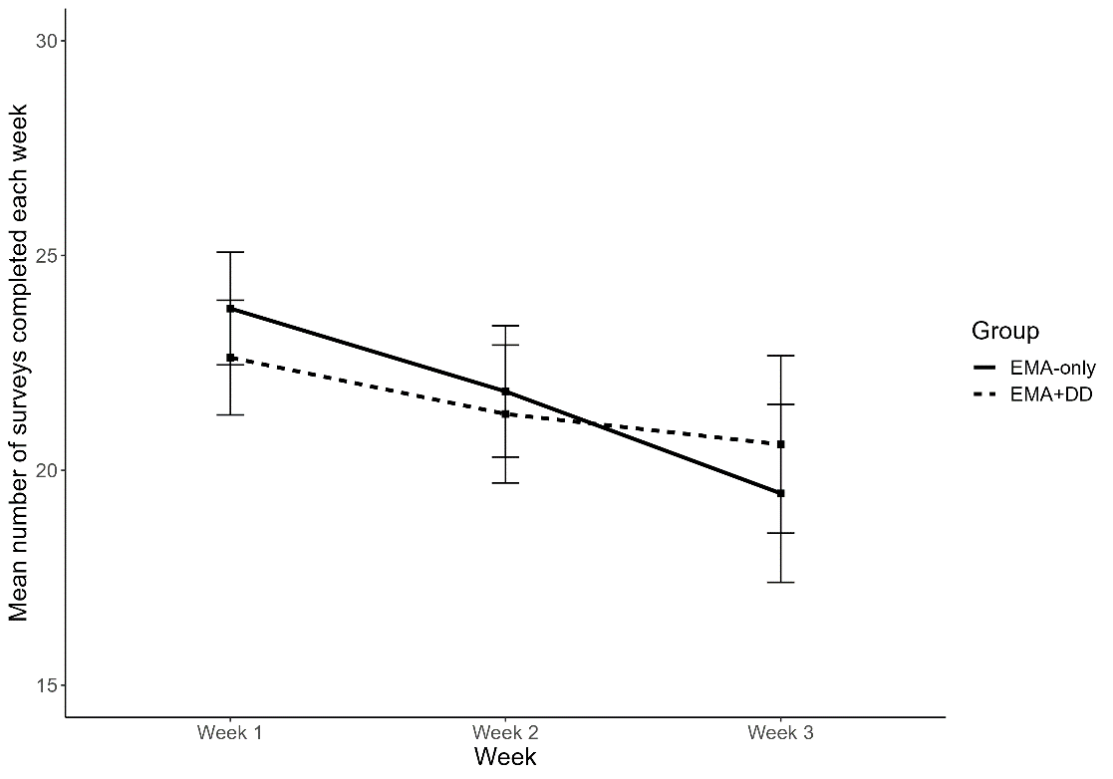
Mean number of surveys completed each week for the EMA+DD group (dashed line) versus the EMA Group (solid line). Mean number completed decreased with each week, with no significant differences between groups. DD: data dashboard; EMA: ecological momentary assessment.

**Figure 3:**
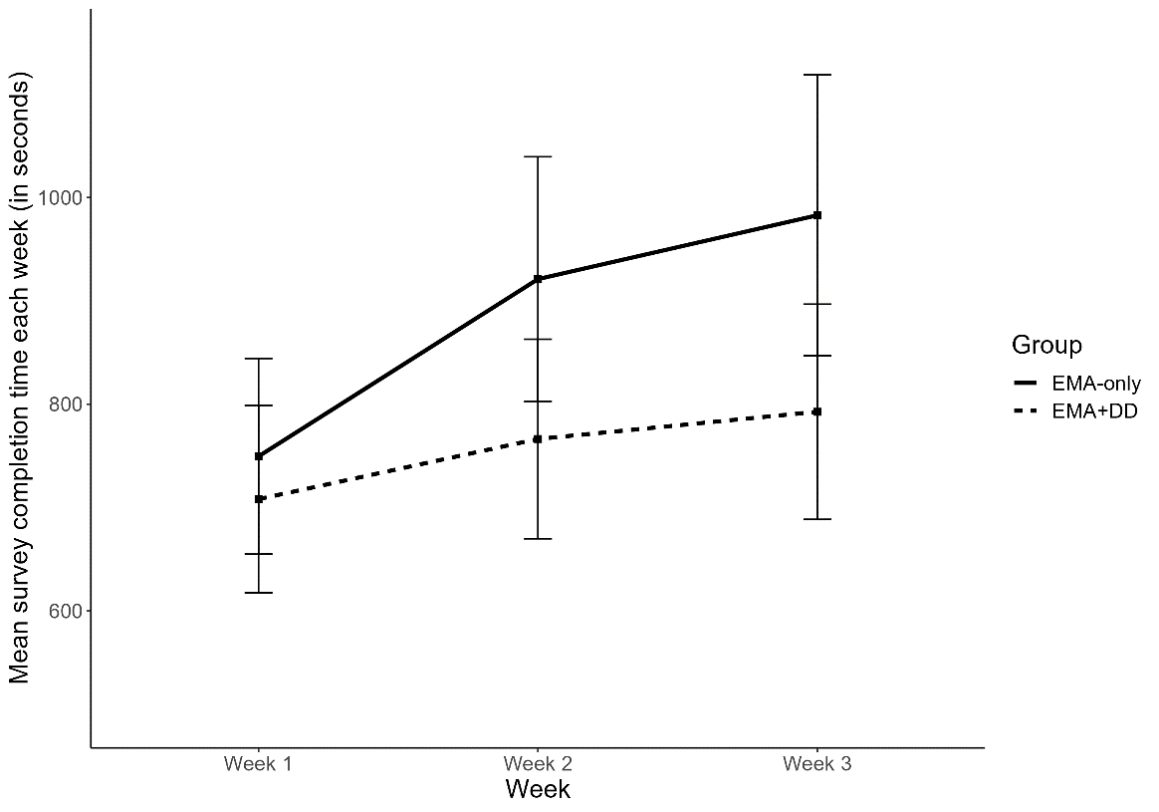
Mean number of seconds to complete a survey for the EMA+ DD group (dashed line) versus the EMA Group (solid line). Survey completion in week 1 was faster compared with later weeks, with the EMA+DD group completing surveys significantly faster overall. DD: data dashboard; EMA: ecological momentary assessment.

For both groups, the mean number of EMAs completed is shown in Figure 2, and the mean number of seconds to complete a survey is shown in Figure 3. A repeated measures ANOVA model showed that overall, there were significant differences in the mean number of surveys completed across the 3 study weeks (*F*_2,178_=17.33, adjusted *P*<.001, η_p_^2^=0.16). The interaction between the groups and study week was not significant (*F*_2,178_=2.41, adjusted *P*=.10, η_p_^2^=0.03). Post hoc analyses also did not reveal any significant differences between the 2 groups at any of the 3 weeks (all *P*>.05; Multimedia Appendix 1).

A separate repeated measures ANOVA showed that there were significant differences in the mean survey completion time across the 3 study weeks (*F*_2,178_=10.96, adjusted *P*<.001, η_p_^2^=0.11). Completion times were faster in the first than in the second and third weeks. Participants in the EMA+DD Group exhibited significantly shorter mean completion times than the EMA Group (*F*_1,89_=3.97, *P*=.049, η_p_^2^=0.04). The interaction between the EMA+DD Group and study week was nonsignificant (*F*_2,178_=2.46, adjusted *P*=.09, η_p_^2^=0.03). Post hoc analyses suggested that there were no differences between the 2 groups at week 1 (t_88.14_=0.63, *P*=.53; *d*=0.13), but there were significant differences and small-to-medium effect sizes in completion time between them at week 2 (t_83.48_=2.01, *P*=.047; *d*=0.42) and week 3 (t_80.85_=2.21, *P*=.03; *d*=0.47; Multimedia Appendix 1).

### Aim 3: Momentary Impact of DD on Compliance for the EMA+DD Group

#### Survey Completion

**Figure 4:**
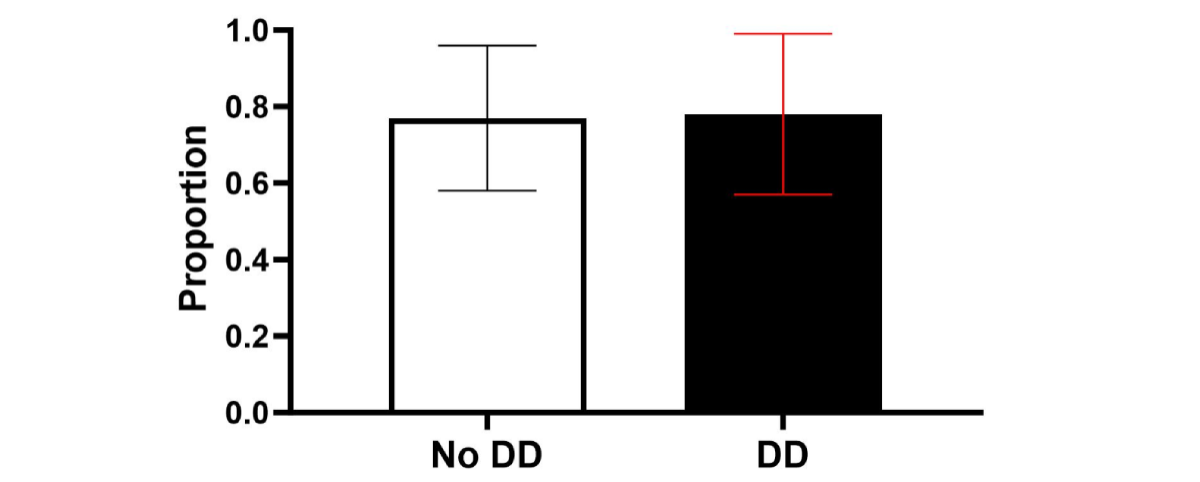
Average proportion of surveys completed by intervention group. DD: data dashboard.

**Figure 5:**
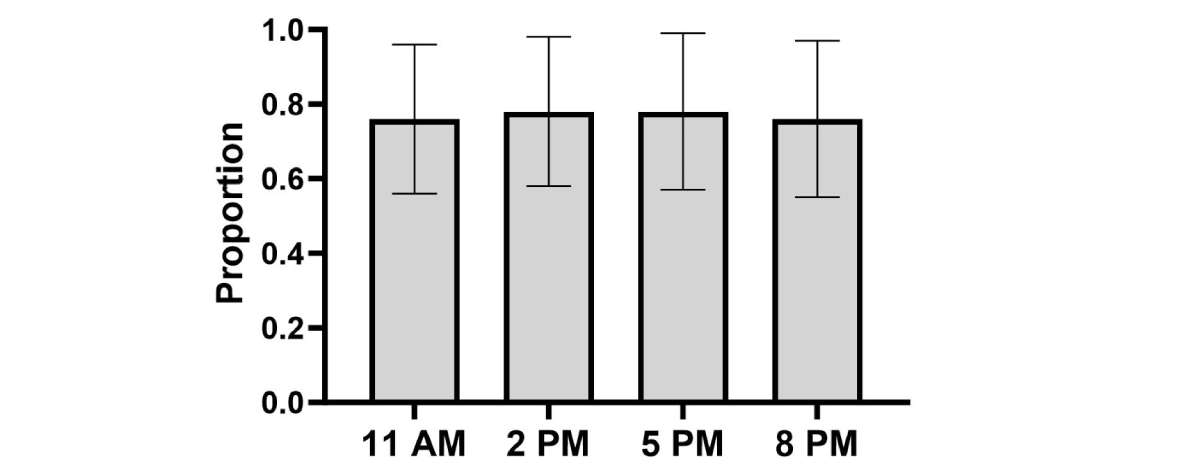
Average proportion of surveys completed across prompt time-of-day.

**Figure 6:**
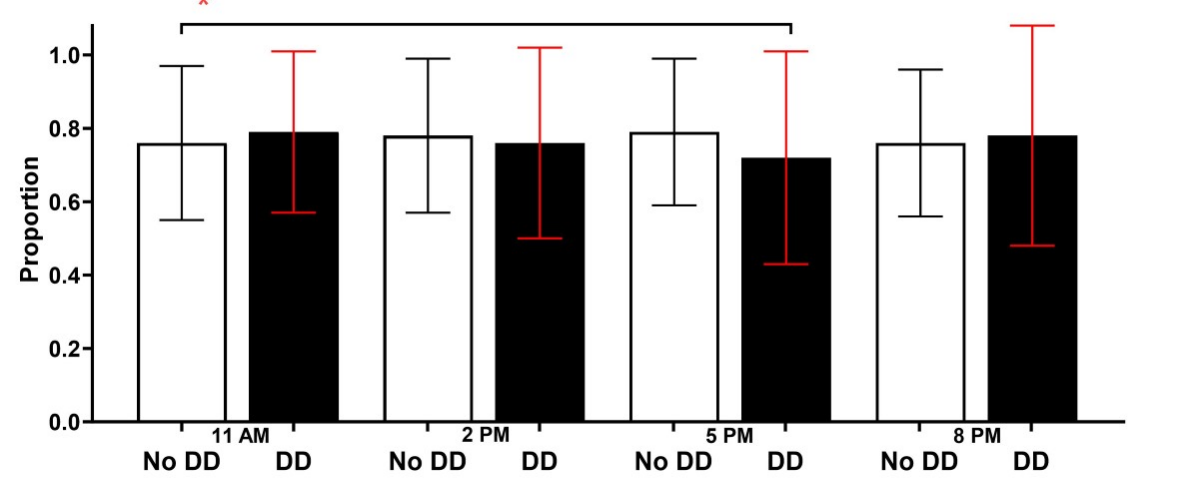
Proportion of surveys completed at each time of day by intervention group. DD: data dashboard. *p<.05.

Across moments in the study within the EMA+DD Group, receiving a DD at a particular prompt did not significantly increase the odds of EMA completion at that same prompt (odds ratio [OR] 1.25, 95% CI 0.94-1.65; *P*=.13; Table 4 and Figure 4). Odds of completing an EMA did not differ significantly by time of day (all *P*>.05; Table 4 and Figure 5). For the DD by time-of-day interaction, the DD was more effective in increasing compliance only when it was sent at 11 AM compared with 5 PM (Table 4 and Figure 6).

**Table 4:** Momentary differences in prompt compliance across all study timepoints for participants in the ecological momentary assessment + data dashboard condition, Model 1.

Model 1 predictors (reference)	Outcome: survey completion within the next hour (n=48, person moments=4014)		
	OR^a^	95% CI	*P* value
(Intercept)	3.07	2.20-4.27	<.001
DD^b^ (none)	1.25	0.94-1.65	.13
2 PM prompt (11 AM)	1.19	0.93-1.52	.16
5 PM prompt (11 AM)	1.24	0.96-1.62	.10
8 PM prompt (11 AM)	1.04	0.82-1.33	.74
DD (none) × 2 PM prompt (11 AM)	0.78	0.54-1.11	.17
DD (none) × 5 PM prompt (11 AM)	0.56	0.37-0.87	.01
DD (none) × 8 PM prompt (11 AM)	0.83	0.54-1.27	.39

^a^OR: odds ratio.

^b^DD: data dashboard.

#### Survey Completion Time

**Figure 7:**
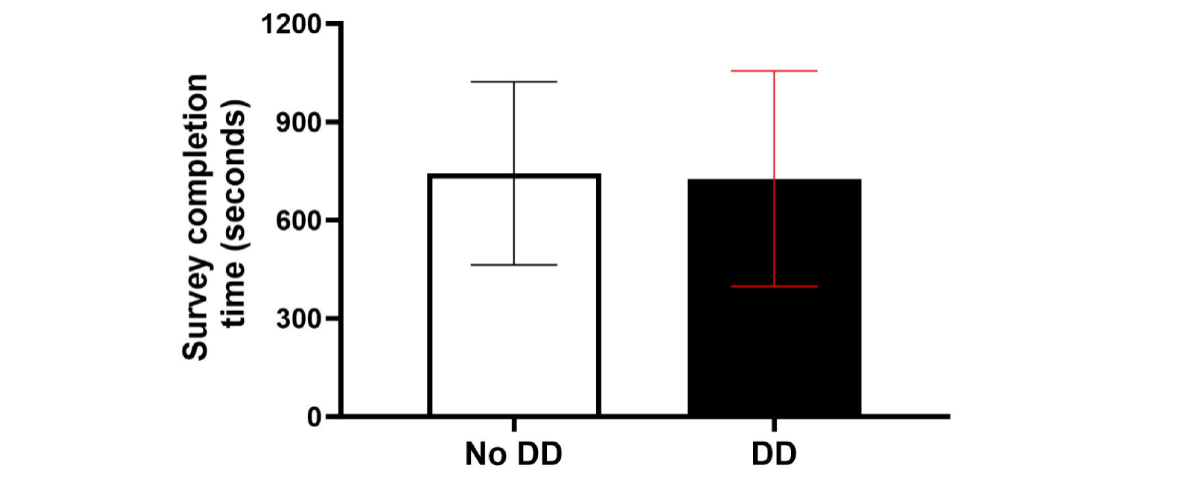
Average survey completion time (and SD) across intervention groups. DD: data dashboard.

**Figure 8:**
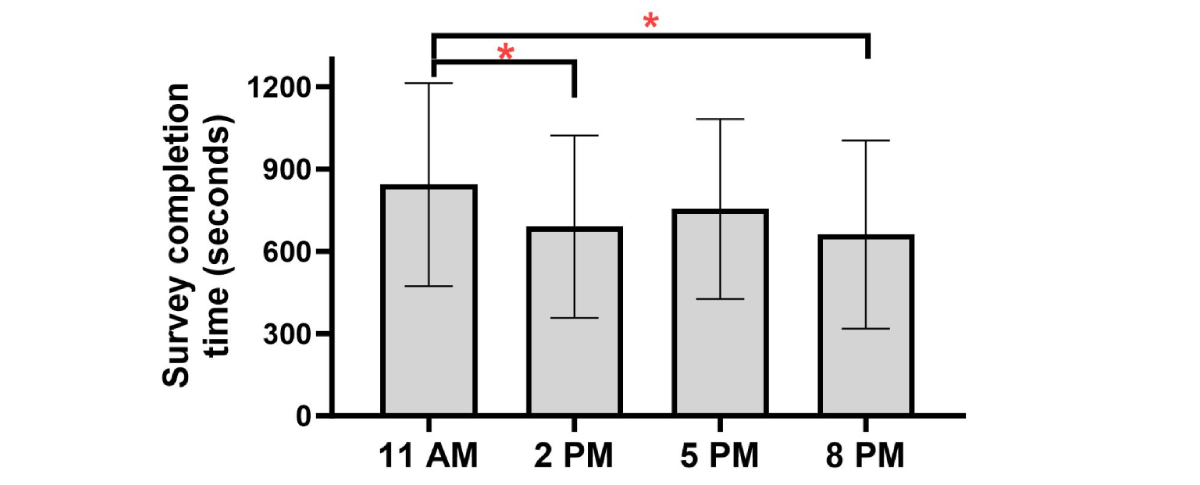
Average survey completion time (and SD) across prompt time-of-day. *p<.01.

**Figure 9:**
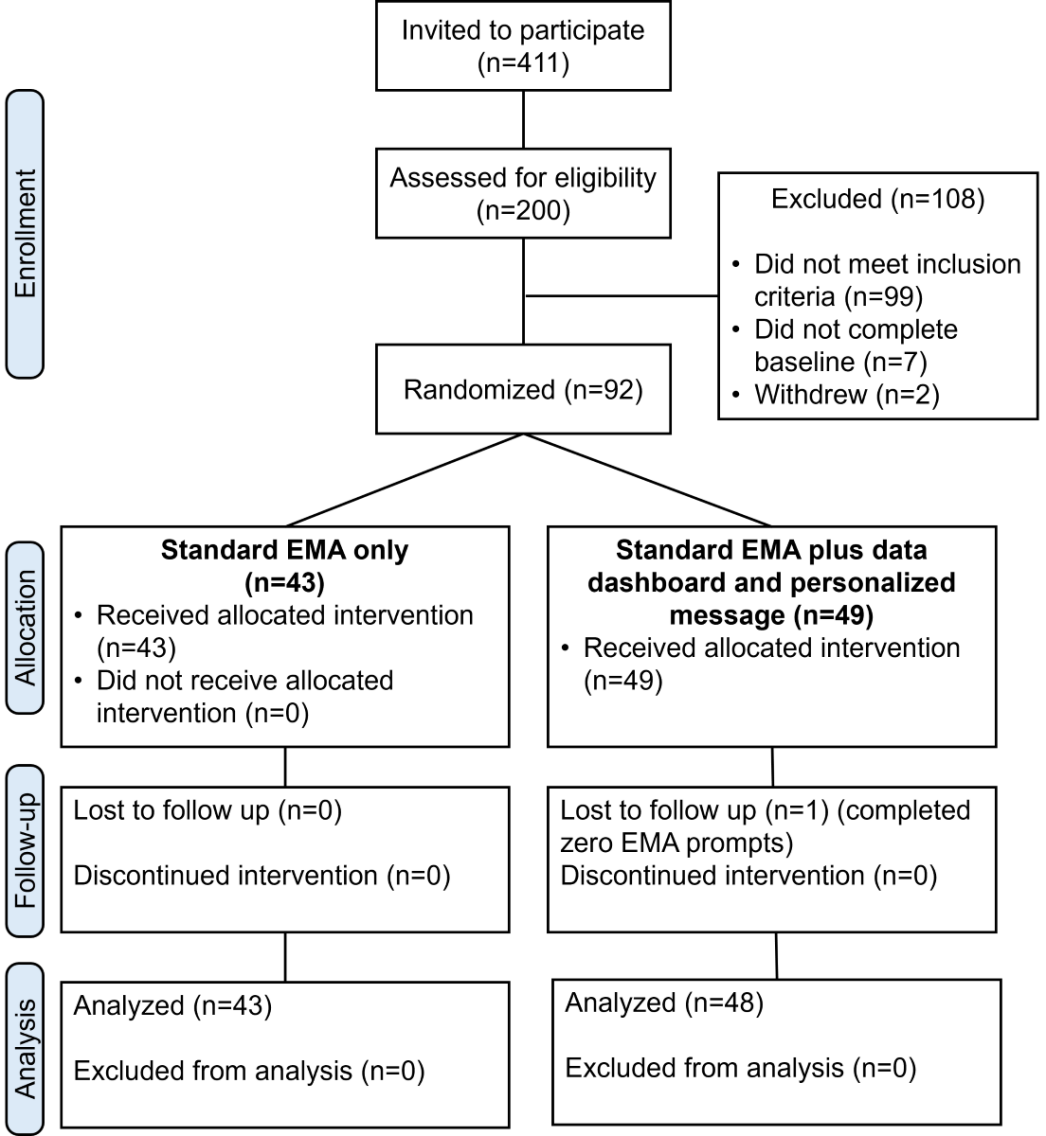
CONSORT (Consolidated Standards of Reporting Trials) flowchart. EMA: ecological momentary assessment.

Across occasions in the study for the EMA+DD Group, providing a DD versus providing no DD did not significantly affect survey completion time (*B*=–86.00, 95% CI –225.50 to 53.60; *P*=.22; and Cohen *d=*–0.10*,* indicating a very small effect size; Table 5 and Figure 7). There were, however, significant momentary differences in completion time based on the time the prompt was delivered. Compared with 11 AM prompts, participants completed EMAs in significantly less time at 2 PM (*B*=–183.80, 95% CI –309.80 to 58.90; *P*=.01; and Cohen *d*=–0.22, indicating a small effect size; Table 5 and Figure 5) and at 8 PM (*B*=–204.20, 95% CI –316.0 to 92.30; *P*<.001; and Cohen *d*=–0.24, indicating a small effect size; Table 5 and Figure 8), but there was not a significant difference compared with 5 PM (*B*=–102.70, 95% CI –227.80 to 22.40; *P*=.10; and Cohen *d*=–0.12, indicating a small effect size; Table 5 and Figure 5). The DD-by-occasion time interaction was not statistically significant (all *P*>.05; Table 5). A CONSORT (Consolidated Standards of Reporting Trials) diagram describing the study procedures is shown in Figure 9.

**Table 5:** Momentary differences in completion time (among completed prompts) across all study timepoints for participants in the ecological momentary assessment + data dashboard condition, Model 2.

Model 2 predictors (reference)	Outcome: moment-level completion time. n=48, person moments=3098			SE		Hoteling		*P* value		Cohen *d*
	*B*	95% CI								
(Intercept)	865.10	744.80 to 985.50	59.50		211.18		<.001		—^a^	
DD^b^ (none)	–86.00	–225.50 to 53.60	69.10		1.55		.22		–0.10	
2 PM prompt (11 AM)	–183.80	–309.80 to 57.90	62.30		8.70		.01		–0.22	
5 PM prompt (11 AM)	–102.70	–227.80 to 22.40	61.90		2.75		.10		–0.12	
8 PM prompt (11 AM)	–204.20	–316.0 to 92.30	55.30		13.62		<.001		–0.24	
DD (none) × 2 PM prompt (11 AM)	83.50	–112.40 to 279.40	96.90		0.74		.39		0.10	
DD (none) × 5 PM prompt (11 AM)	48.70	–178.00 to 275.40	112.20		0.19		.67		0.06	
DD (none) × 8 PM prompt (AM)	53.00	–129.70 to 235.80	90.40		0.34		.56		0.06	

^a^Not applicable.

^b^DD: data dashboard.

## Discussion

The overall goal of this pilot study was to examine the impact of providing a real-time DD displaying participants’ progress in an EMA study on multiple indicators of compliance and subjective experiences with the EMA protocol. Our pilot had 2 randomly assigned groups of college students who recently engaged in alcohol and cannabis use: the EMA Group completed a 21-day EMA protocol on health behaviors, and the EMA+DD Group completed the same 21-day protocol but also received personalized messaging and a DD at 1 of 4 prompts each day. Overall, between-group findings and week-level analyses indicated that the groups did not differ in the number of EMAs completed. Thus, personalized dashboards may not promote greater EMA compliance. One explanation for this finding is the DDs in our study were relatively simple in their personalizations. Participants received DDs displaying how they were progressing in the study, but messaging could include greater integration of personal preferences for certain framing of messages (eg, motivational and humor), greater interactive components, and inclusions of the different types of data people prefer to have displayed (eg, 1 participant preferred not to know the number of days remaining in the study). A lack of effect of greater EMA compliance from the DDs, however, might be best understood alongside other aspects of our study results, including differences between groups in the speed of completing the EMAs. We found that participants in the EMA+DD group completed their EMAs quicker than the EMA Group, which did provide some indication that perhaps the DD played a role in engagement. One reason for the potential increase in engagement is that because compliance was generally quite high regardless of group, it may have been challenging to detect a larger difference between the groups in compliance rates. Quicker completion time may have been an outcome that could be detected among the many participants who would have completed their EMAs regardless of whether they were in the intervention group. Faster completion of EMA prompts could be useful as it minimizes the amount of time between having an acute experience (eg, brief emotional episode) and recalling that experience via self-report. However, quicker completion time in terms of reductions in minutes or even seconds may not add value for recalling certain experiences or behaviors (eg, whether one was at a bar in the hours prior).

Importantly, although compliance rates overall declined across the 3 weeks, in the last 2 weeks participants in the EMA+DD Group responded significantly faster than the EMA Group. This is a key finding, as it is common for participants to habituate to interventions during the course of a study, resulting in diminishing effects over time [23]. Further, given general compliance concerns [9] and the tendency for study compliance to wane over time in EMA protocols [24], providing a personalized message and DD appears to be an effective way to increase completion times, particularly in the later weeks of a study. Our results parallel other microrandomized studies in that intervention effects often depend on the length of time in the study. For example, Carpenter et al [15] found that different messages to enhance self-monitoring of alcohol use varied in their effectiveness early versus later in the study.

Differences were also observed with regard to subjective experiences, such that participants who received a DD were more likely to perceive the text messages as personalized, and—although nonsignificant—observed small-to-medium effect sizes that the EMA + DD group viewed the compensation level as adequate and the protocol as less burdensome. Qualitative findings were generally in line with these results, with participants indicating that seeing their progress helped them stay more engaged. Further, participants seemed to be particularly motivated to secure the bonus. The most commonly reported areas of needed improvement were a clearer explanation of the wheel and wanting full access to the DD at all times. Future work may build from these findings by providing a web-based digital module that provides an overview of the DD to participants. The finding that participants wanted access to the DD throughout the study is encouraging, as it suggests that participants did not feel burdened by frequently viewing their DD. This finding is in alignment with users’ preferences for personalization pertaining to ongoing monitoring and feedback during mobile health interventions [25].

Interestingly, within the EMA+DD Group, providing a DD did not significantly impact participants’ likelihood of completing the survey or completion time at the moment-level. There was, however, a significant moderation effect based on the time of day the DD was sent. Specifically, the impact of a DD on the likelihood of completing the survey was greater at the first prompt of the day (11 AM) than at the 5 PM prompt. Perhaps the momentary effects of a DD are subtle and depend on time-varying moderators (eg, time of day, time in the study) to increase momentary effectiveness, although this finding will need to be replicated to draw firm conclusions about the effectiveness of DD given the time of day they are sent. One possibility is that because the 11 AM survey was the first in the series within the day, participants may experience a “Zeigarnik effect” in which participants remember unfinished or interrupted tasks more than completed tasks [26]. Our overall findings, however, suggest that there may be some subtle benefits from delivering a DD (ie, greater completion speed, and prompt completion at certain times of day). Future research may benefit from exploring whether having access to the dashboard throughout the study period versus receiving the DD at particular times each day is more impactful on compliance. Future research may benefit from exploring whether having access to the dashboard throughout the study period versus receiving the DD at particular times each day is more impactful on compliance.

Several limitations should be noted. First, participants were college students who were primarily White from a large, public university in the Northeastern region of the United States. Findings may be less generalizable to young adults who do not attend college or college students who are from underrepresented backgrounds. It is unclear to what extent age or college-enrollment status impacted study findings particularly engagement and survey completion time; future research exploring the impact of DDs on engagement and compliance patterns from a more diverse and wider age range would be highly beneficial. Second, relatedly, participants were recruited from a larger study based on their recent alcohol and cannabis use, and were thus a convenience sample and not necessarily representative of college students at the university. Third, completion time was used as a metric of compliance, which may be impacted by individual differences in completing surveys or unknown day-level factors. Researchers may aim to use software that permits the duration of time between receiving the prompt and initiating the survey to be accurately assessed. This will likely serve as a more precise measure of completion speed. Fourth, although the majority of students at the present institution own an iPhone, an estimated 20% of students use an Android and were unable to participate in this study. Future work would benefit from building a mobile app or using other software accessible for both Android and iPhone. Last, given that this study was a small pilot study, we were underpowered to detect significance for some small effects, including the effect of person-level factors and a more thorough investigation into time-varying moderation effects. This was compounded by a large number of exploratory hypotheses considered in this pilot study. Future work should build from these findings with a larger, more diverse sample of young adults to replicate and clarify the utility of DDs more broadly.

Overall, this study found that providing participants with a DD resulted in faster completion times, particularly in the later weeks of the EMA protocol. Findings may have implications for future EMA studies and for both microrandomized trials and just-in-time interventions that rely heavily on participant compliance with self-report surveys. Specifically, given that participants overall liked having access to the DD and exhibited faster completion rates, providing participants with a DD may be highly useful for engagement, particularly for researchers aiming to assess behavior that occurs close in time to when the survey prompt is provided. Faster completion times would be particularly critical when survey responses are used to trigger the just-in-time delivery of intervention content. Although future research is needed to determine whether constant access to a DD or time-specific access is more effective, our study suggests that providing a DD earlier in the day or at the first prompt of the day is more beneficial than later in the day. Such an approach may be particularly useful for daily diary studies that inquire about yesterday’s behavior early in the day.

## Appendix

Multimedia Appendix 1 Group differences in study compliance and survey completion time assessed at the week level.

## Abbreviations

CONSORT: Consolidated Standards of Reporting Trials
DD: data dashboard
EMA: ecological momentary assessment
OR: odds ratio
REDCap: Research Electronic Data Capture
