# Supplementary material for: Impact of Providing a Personalized Data Dashboard on Ecological Momentary Assessment Compliance Among College Students Who Use Substances: Pilot Microrandomized Trial
Source: JMIR Form Res. 2024 Dec 5;8:e60193. doi: 10.2196/60193 (PMC11659699; doi:10.2196/60193)
Supplement: Multimedia Appendix 1 [file formative_v8i1e60193_app1.docx]

**Multimedia Appendix 1.** Group differences in study compliance and survey completion time assessed at the week level.

| Outcome | | EMA-Only  Group  *M* | EMA + DD Group  *M* | *t* | *df* | *p-*value | *Cohen’s d* |
| --- | --- | --- | --- | --- | --- | --- | --- |
| Compliance | Week 1 | 23.77 | 22.63 | 1.213 | 88.86 | 0.228 | 0.25 |
|  | Week 2 | 21.84 | 21.31 | 0.470 | 88.99 | 0.639 | 0.10 |
|  | Week 3 | 19.47 | 20.6 | -0.774 | 88.69 | 0.441 | -0.16 |
| Speed of Survey Completion | Week 1 | 749.58 | 708.13 | 0.628 | 88.14 | 0.531 | 0.13 |
|  | Week 2 | 921.01 | 766.32 | 2.013 | 83.48 | 0.047 | 0.42 |
|  | Week 3 | 982.76 | 792.68 | 2.206 | 80.85 | 0.030 | 0.47 |
